# Dr. Eckermann on Osmosis and Dental Caries*“Dental Caries in Relation to Oral Osmosis.” Carl Bloms, Lund. London: Williams & Norgate, 11 Henrietta Street, W.C. 2.

**Published:** 1920-02

**Authors:** William Rushton


### DR. ECKERMANN ON OSMOSIS AND DENTAL CARIES*

BY WILLIAM RUSHTON, L.D.S.

Within the last few years the conclusions arrived at by the epoch-marking and laborious researches of Miller on the origin of dental caries, have been attacked by Dr. Ragnar Eckermann, of Malmoe, who some time ago contributed a series of articles to this journal, in which he sought to prove that caries was caused, not by lactic acid, but by the action of osmosis. Let us briefly summarize what is meant by osmosis. If a solution of sugar be enclosed in a vessel having semi-permeable walls—*i. e.,* permeable by the water, but not by the dissolved sugar—and then this vessel be placed in water, it will be found that some of the water diffuses through the membrane into the solution and continues until a certain pressure is attained; this is termed the osmotic pressure. Dr. Eckermann's

*"Dental Caries in Relation to Oral Osmosis." Carl Bloms, Lund. London: Williams & Norgate, 11 Henrietta Street, W.C. 2.

argument is that, owing to the large quantities of condiments (especially salt and sugar) consumed by the more civilized nations, the saliva is frequently loaded with dissolved material heavier than the blood-plasm in the tooth pulp. He maintains that dentine is a semi-permeable membrane and that when the saliva is in the aforesaid condition, the blood-plasm of the pulp is drawn or forced up towards the tooth surface. Perfectly sound enamel is impermeable, but in the large majority of teeth there are slight histological defects, and at these points the dentine can act as a semi-permeable membrane, as it also can at the neck of the tooth where no longer shielded by the gum. At these weak points Dr. Eckermann affirms that osmosis takes place and the blood-plasm is forced up the dentine, forming a faint rose-colored streak or tract which he designates the "caries canal." It will be seen that his theory directly conflicts with that of Miller, who gives a chemical solution as the first step, starting from the tooth surface, while Eckermann gives a physiological action starting from the tooth pulp. These "caries canals" are plainly visible to the naked eye in sections of teeth slightly decayed (he gives photographs of several), and he claims that the rose color is from the blood and that he has conclusively proved the presence of iron in them, which is found in the blood. He goes on to say: "The destructive process of the caries canals is caused by an invasion of micro-organisms probably assisted by strong acidity from mineral acids." Dr. Eckermann does not state how the micro-organisms commence their invasion, or why. Is it because they receive nutriment from the extruded blood-plasm, or is it because the dentine of the "caries canal" is of lower resisting power? We consider that this point has not received the attention in his work that it demands, and we should be glad to hear what he has to say on the matter, especially as he affirms that carious dentine must be regarded as a transformed tissue, which transformation involves a protection of the body against the invasion of foreign materials.

Dr. Eckermann considers that the action of all organic acids on the teeth is negligible and, as regards lactic acid, prolonged retention of teeth in it, even when it is frequently renewed, produces practically no effect on the enamel. Any action in the mouth in presence of the saliva is, to him, unthinkable, in addition to which he considers it absurd that milk and carbohydrates, which have been used as the food of man since the days of Pithecanthropus, should in these last few years be suddenly suspected. If any acid does destroy the teeth, he says, it is probably hydrochloric acid, obtained from the large quantity of salt now used both in fresh and preserved foods, which comes into the mouth partly from the gastric juice and partly a product of catalysis.

As regards Dr. Stanley Colyer's "Sugar Theory," Dr. Eckermann considers Dr. Colyer correct as to the agent, but wrong as regards the way it works, as it is a very weak acid-former, and that his theory is in reality based upon the carbohydrate theory of Miller with the Sim Wallace variation.

Some of the statements which Dr. Eckermann brings against the Miller theory are as follows: No one has yet found a microbe to which caries can be positively ascribed. Pickerill has found no difference bacteriologically between the mouths of European children with carious teeth and Maori children with perfect teeth. Michaels states that the most active caries are found in those individuals who are hypo-acid rather than hyper-acid. "The consequences of Miller's theory are that caries is preventable by cleanliness and antiseptics. The experience of several decades shows the opposite, and we know that our most diligent and most frequent patients are fanatical mouth hygienists." The Third International Dental Congress held in Paris passed the following resolution: "Our present knowledge of dental caries can not explain the different forms of this malady. It is assumable that caries come from the inner parts of the tooth and grow outwardly."

Dr. Eckermann has attacked the problem from the standpoint of the chemist and physicist rather than that of the microscopist, and his numerous experiments are mostly on these lines. Two sets of his microphotographs, however, are deserving of mention, one set showing two sections of carious dentine in which red blood corpuscles are seen. Although the red corpuscles are much greater in size than the tubules, he affirms that they are capable of being carried through these owing to their elasticity and to their shrinking under osmotic influence. Two others are given of a section of a sound tooth injected before extraction for regulation. Under a local anaesthetic the pulp was drilled into and injected with fuchsine. After twenty minutes the tooth was extracted after the needle had been broken off and the bore-hole with the part of the needle left in sealed with wax. The apex was also sealed and the tooth immersed for twenty-four hours in Ringer's solution, taken out, cut into very thin sections and embedded in Canada balsam. Many such experiments were made, the younger the tooth the better the results, which were that the stain had been conveyed to the dento-enamel junction. The author's conclusions were that the color had reached the interglobular spaces not directly from the bore-hole, but from the pulp through the tubules, showing that there is a circulation of fluid from the pulp cavity to the interglobular layer.

To criticize faithfully a work of this kind requires more knowledge of chemistry, physics and histology than the present writer can lay claim to. But Dr. Eckermann's work has been so original, serious and painstaking that these few lines are written to appreciate the fact that he has gone to the trouble and expense of writing it in English (so that it may be read and criticized by the English-speaking world) and publishing it in Sweden, because, owing to the war, it was impossible to get the work printed in England. Whether he is right or wrong in his theories, it is to be hoped that his work will not be treated with coldness or, still worse, with silence, but that some competent worker will take the trouble to investigate his conclusions and give the profession the benefit of the result.

Sometimes, owing to writing in a language not his own, Dr. Eckermann is somewhat obscure, and sometimes apt to repeat himself, but on the whole he is to be congratulated on the way he has laid his theories before the profession. Whether he will succeed in convincing the profession is another matter. It is not easy to believe that a harmless fluid like blood-plasm, even if it is acted on by osmotic influence, can do any harm, and we should like to be informed how it does it.—*Dental Record.*

